# Comprehensive Analysis of Ferroptosis Regulators in Lung Adenocarcinomas Identifies Prognostic and Immunotherapy-Related Biomarkers

**DOI:** 10.3389/fmolb.2021.587436

**Published:** 2021-03-12

**Authors:** Sijin Sun, Wei Guo, Fang Lv, Guochao Zhang, Juhong Wang, Renda Li, Fengwei Tan, Ning Li, Qi Xue, Yibo Gao, Shugeng Gao, Jie He

**Affiliations:** ^1^ Department of Thoracic Surgery, National Cancer Center/National Clinical Research Center for Cancer/Cancer Hospital, Chinese Academy of Medical Sciences and Peking Union Medical College, Beijing, China; ^2^ Clinical Trials Center, National Cancer Center/National Clinical Research Center for Cancer/Cancer Hospital, Chinese Academy of Medical Sciences and Peking Union Medical College, Beijing, China; ^3^ State Key Laboratory of Molecular Oncology, National Cancer Center/National Clinical Research Center for Cancer/Cancer Hospital, Chinese Academy of Medical Sciences and Peking Union Medical College, Beijing, China

**Keywords:** lung adenocarcinoma, ferroptosis, prognosis, immunotherapy, machine learning

## Abstract

Ferroptosis is a newly discovered type of programmed cell death that differs from canonical apoptosis. However, the potential role of ferroptosis in lung adenocarcinoma (LUAD) has not been elaborated. In total, 1,328 samples from databases and 36 ferroptosis regulators were included in this study. By combining random survival forest and principal component analysis algorithms, a robust prognostic ferroptosis-related risk score (FRRS) was constructed, and the performance was validated in three independent datasets. Based on the median risk score, two subgroups were identified. Then, comparisons, including of mutational profiles, functional enrichment analyses and immune components, were conducted between subgroups. An immunotherapy cohort was applied to explore potential therapeutic-related biomarkers. Finally, the clinical utility of FRRS was validated in a proteomic cohort. In the TCGA-LUAD cohort, FRRS was calculated using the expression of 11 selected genes, and patients with high FRRS had a significantly (*p* < 0.001) worse prognosis than those with low FRRS. Multivariate regression suggested that FRRS was an independent prognostic factor. Functional enrichment analysis indicated that FRRS was mainly involved in cell cycle, metabolic and immune-related pathways. Furthermore, FRRS was shown to be significantly (*p* < 0.001) associated with the abundance of CD8 T cells and tumor mutation burden (TMB). The combination of TMB and FANCD2 expression, the main contributor to FRRS, substantially increased the precision of predicting the therapeutic response. In conclusion, the present study revealed the potential role of ferroptosis regulators in LUAD and identified ferroptosis-related biomarkers for prognostic and immunotherapeutic predictions.

## Introduction

Cancer is a major public health problem worldwide. Lung cancer remains the most common cancer, with the highest morbidity and mortality according to the latest cancer statistics (Siegel et al., 2020). Among all subtypes of lung cancer, nonsmall-cell lung cancer (NSCLC), including adenocarcinoma, squamous cell carcinoma and large cell carcinoma, accounts for approximately 85% of all cases (Bade and Cruz, 2020). Currently, in most countries, lung adenocarcinoma (LUAD) has surpassed squamous cell carcinoma as the major type of NSCLC and represents more than one-third of total cases (Bade and Cruz, 2020). Despite great progress in targeted therapy and immunotherapy, the overall 5 years survival rate in men and women remains 57% for patients with stage I disease and declines to 4% for those with stage IV disease (Miller et al., 2019). The primary treatment of early-stage LUAD is surgical resection, while targeted or systemic therapy is applied for advanced or metastatic disease. Recently, KEYNOTE-042 investigators announced that pembrolizumab outperformed conventional chemotherapy in advanced patients regardless of the PD-L1 tumor proportion score (Mok et al., 2019). Nevertheless, due to the heterogeneous nature of cancer genomes, a significant portion of patients did not benefit from immunotherapy. Further study of the mechanisms by which immune components can be regulated to improve immunotherapeutic efficacy in LUAD is crucial. It is also critical to identify reliable biomarkers that can predict therapeutic response.

Ferroptosis, distinct from apoptosis, is a recently coined form of programmed cell death activated by iron-dependent peroxide lipid accumulation (Dixon et al., 2012). It is a complex and vulnerable process affected by a variety of crosslinks among lipids, iron, amino acid metabolism and environmental stress (Galluzzi et al., 2018; Hirschhorn and Stockwell, 2019). Although the physiological role and function of ferroptosis in normal mammalian development have not been fully elucidated, emerging evidence has revealed that ferroptosis is implicated in a number neoplastic contexts, including breast cancer, renal cell carcinoma and prostate cancer (Lue et al., 2017; Hultsch et al., 2018; Tousignant et al., 2020). Recent studies have suggested that ferroptosis may play a role in tumor suppression through metabolic regulation and promotion of cell death (Gao et al., 2015; Jiang et al., 2015). In addition, *in vitro* studies illustrated that the ferroptosis process is modulated by the tumor suppressor TP53 via its transcriptional targets (Jiang et al., 2015). With regard to clinical application prospects, it has been confirmed that chemotherapy-resistant cancer cells are vulnerable to ferroptosis induced by the lipid peroxidase pathway, which represents a novel therapeutic direction for the treatment of drug-resistant cancer (Hangauer et al., 2017; Viswanathan et al., 2017). Of particular importance, in the context of the development of immunotherapy, research on enhancing immunotherapy efficacy has become a hotspot. Wang et al. uncovered that agents that induce ferroptosis can improve the immunotherapeutic effect by promoting CD8^+^ T cells to trigger ferroptosis (Wang et al., 2019). A deeper comprehension of the mechanisms and functions of ferroptosis will pave the way for developing novel cancer therapeutic strategies.

Previous *in vitro* and *in vivo* studies have shown that dysregulation of ferroptosis may play a critical role in drug resistance and immune evasion in tumors (Angeli et al., 2019). To the best of our knowledge, there has been no comprehensive analysis of ferroptosis regulators in LUAD. In the present study, the expression profile and copy number variation of 36 ferroptosis regulators were first described in detail using The Cancer Genome Atlas (TCGA) database. Next, a ferroptosis-related prognostic model was constructed in the TCGA-LUAD cohort and validated in three independent cohorts and a proteomics cohort. Then, different genomic mutation profiles and biological functions and pathways related to this model were explored. Finally, correlation analyses between risk score and immune components were conducted, and three ferroptosis regulators were identified as potential biomarkers for predicting immunotherapeutic response, providing a basis for future implementation of ferroptosis in the clinical application of treating LUAD.

## Method

### Data Acquisition and Preprocessing

The training cohort contained 500 LUAD patients with both RNA-seq and clinical information extracted from the TCGA-LUAD project database (https://portal.gdc.cancer.gov/). Gene expression quantified with fragments per kilobase million (FPKM) was first downloaded from the database and transformed into transcripts per million (TPM) before further analysis. Three independent validation cohorts (GSE3141, GSE30219, GSE31210) were obtained from the Gene Expression Omnibus (https://www.ncbi.nlm.nih.gov/geo) by the GEO query R package (Bild et al., 2006; Davis and Meltzer, 2007; Okayama et al., 2012; Rousseaux et al., 2013). For microarray datasets, probe IDs were first converted into gene symbols, and genes that corresponded to multiple probes were collapsed using mean values. Logarithmic transformation and standardization were then performed for each gene. IMvigor210 is an immunotherapy cohort, including 348 metastatic urothelial cancer (mUC) patients treated with atezolizumab. A fully documented package named IMvigor210CoreBiologies provided the methods and processed data for this cohort (Mariathasan et al., 2018). RNA-seq data, as count values, were first normalized to the trimmed mean of M-values (TMM) in *edgeR* R package and then transformed by voom in the *limma* R package (Smyth, 2005; Robinson et al., 2010). Only cases with complete gene expression and clinical information were included in the further analysis. Gene-level mutation (VarScan) and copy number data of the TCGA-LUAD cohort were acquired from the TCGA data portal (https://portal.gdc.cancer.gov/). In terms of copy number variation, the GISTIC2 approach was implemented to quantify gene copy gain and loss (Mermel et al., 2011). A detailed description of the included cohorts is shown in Table 1.

**TABLE 1:** Description of lung adenocarcinoma cohorts used in this article.

Citation	Source	Accession	Platform	Number of cases
−	TCGA-LUAD	−	IlluminaHiSeq	500
Bild et al. (2006)	GEO	GSE3141	GPL570	58
Rousseaux et al. (2013)	GEO	GSE30219	GPL570	85
Okayama et al. (2012)	GEO	GSE31210	GPL570	226
Mariathasan et al. (2018)	IMvigor210	−	IlluminaHiSeq	348
−	CPTAC-LUAD	−	Proteome	111

### Construction and Validation of a Ferroptosis-Related Prognostic Model

The list of genes involved in ferroptosis was retrieved from a recent review on this topic (Stockwell et al., 2017). Overall, 36 ferroptosis regulators were included in the present study. To construct a robust ferroptosis-related prognostic scoring system, the random survival forest algorithm was first applied to reduce the dimension of features, and gene selection was performed according to both variable importance (VIMP) and minimal depth (Ishwaran et al., 2008). Only genes that ranked in the top 15 lists for both VIMP and minimal depth were selected. Then, principal component analysis (PCA) was performed, and principal components with the most significant prognostic values calculated by Cox regression were extracted for use as a ferroptosis-related risk score (FRRS) (Wold et al., 1987). The performance of FRRS was first evaluated in the TCGA-LUAD cohort and then validated in three independent LUAD cohorts from GEO. Then, differential expression analysis between tumor and normal tissue was conducted on selected genes. Finally, associations between gene expression and copy number variation of these selected genes were explored. Samples in TCGA-LUAD were divided into high-risk and low-risk subgroups according to the median risk score for further analyses.

### Molecular Characteristics of Genes in the Ferroptosis-Related Risk Score

To further elaborate the basic characteristics of selected genes, differentially expressed gene (DEG) analyses between tumor and normal tissue were first conducted in the TCGA-LUAD cohort. Then, correlation analyses between copy number variation defined by the GISTIC2 approach and gene expression defined by TPM were performed among these genes.

### Genomic Characteristics and Tumor Mutation Burdens Between Different Risk Subgroups

To evaluate the differences in genomic alternations among risk subgroups, the mutation profile and mutation type of the top 15 genes with the highest mutation frequency were drawn in oncoplots (Mayakonda et al., 2018). Tumor mutation burden (TMB) was defined as the total number of nonsynonymous mutations in a patient’s whole exome. In the present study, TMB was calculated as the number of nonsynonymous mutations per mega-base (using 38 Mb as the size of exome). After TMB estimation for each case, correlation analysis between TMB and FRRS was applied.

### Pathway Enrichment Analysis of DEGs Between Subgroups

To explore the potential biological functions and pathways related to FRRS, DEG analysis was performed using the *edgeR* R package between subgroups. A cutoff of adjusted *p* < 0.01 and |log_2_ (fold change)| > 2 were used to define DEGs. The top 100 significant DEGs ranked by adjusted *p* value were selected. Then, GO biological process and KEGG pathway analyses were applied to identify the potential functions of these DEGs using the *clusterProfiler* R package (Yu et al., 2012). Benjamini-Hochberg (BH) adjustment was used to calculate adjusted *p* values.

### Gene Set Enrichment Analysis of FRRS

Gene set enrichment analysis (GSEA) is an approach to identify specific pathways or processes that are overrepresented in predefined subgroups, which is an alternative to DEG-based functional analysis (Subramanian et al., 2005). Curated gene sets were downloaded from the Molecular Signatures Database v7.1 and included for analysis. The BH method was applied to adjust for multiple testing, and permutations were set to 1,000.

### Exploring Relationships Between Immune Components and FRRS

To verify the potential relationship between FRRS and immune components, both emerging immunotherapy targets and infiltrating immune cells were included. The list of potential drug targets involved in innate and adaptive immune processes was extracted according to a recent review (Burugu et al., 2018). Comparisons of target gene expression between different risk groups were conducted. In terms of immune cells, a computational algorithm named ImmuCellAI was utilized to estimate the abundance of 24 infiltrating immune cell types based on transcriptome data (Miao et al., 2020). Associations between *FRRS* and infiltrating immune cell abundances were analyzed by Pearson correlation.

### Exploring the Potential in Clinical Immunotherapy Response Prediction

To identify markers with predictive value in the immunotherapy response, the top three ferroptosis regulator genes (CISD1, FANCD2, and SLC3A2) that contributed to the FRRS were extracted, and association and Kaplan-Meier survival curve analyses were performed using these genes in the IMvigor210 cohort. A receiver operating characteristic (ROC) curve was applied to assess the predictive efficacy of gene expression for the *immunotherapy response.*

### Protein-Level Validation of FRRS and Selected Ferroptosis Regulators

For further validation of the clinical utility of FRRS and selected ferroptosis regulators, an independent proteomic LUAD cohort, including 111 cases, was obtained from The Clinical Proteomic Tumor Analysis Consortium (CPTAC) (https://proteomics.cancer.gov/programs/cptac). An unshared log ratio was used for quantification of protein expression. Comparisons of selected ferroptosis regulators between normal lung tissue and LUAD were performed. The prognostic value of FRRS was also evaluated in this cohort. Furthermore, immunohistochemical staining images were extracted from The Human Protein Atlas project (https://www.proteinatlas.org/) to verify the true existence of high expression.

### Statistical Analysis

For comparisons of means between two groups, the Wilcoxon signed-rank test was used, and the Kruskal-Wallis test was applied for comparison of means of more than two groups. The chi-square test was performed to test categorical data. Regarding survival analysis, both the Kaplan-Meier survival curve and Cox regression model were implemented. To choose the best cutoff value for subgroup stratification, the R package “maxstat” was applied, providing a cutoff value that corresponded to the factor most significantly related to survival. In the current article, the median was selected as the indicator for group stratification, except for DEG analysis, which used quartile grouping instead. The area under the curve (AUC) was calculated from the ROC curve using the *pROC* R package (Robin et al., 2011). All statistical analyses were carried out using R version 3.6.2. *p* < 0.05 was considered statistically significant unless otherwise specified.

## Results

### Construction of a Ferroptosis-Related Prognostic Model in TCGA-LUAD

**FIGURE 1:**
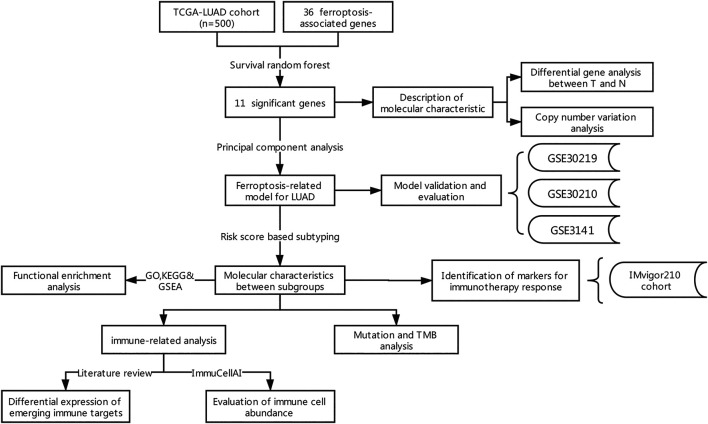
Flowchart of the comprehensive analysis of ferroptotic regulators in LUAD.

**FIGURE 2:**
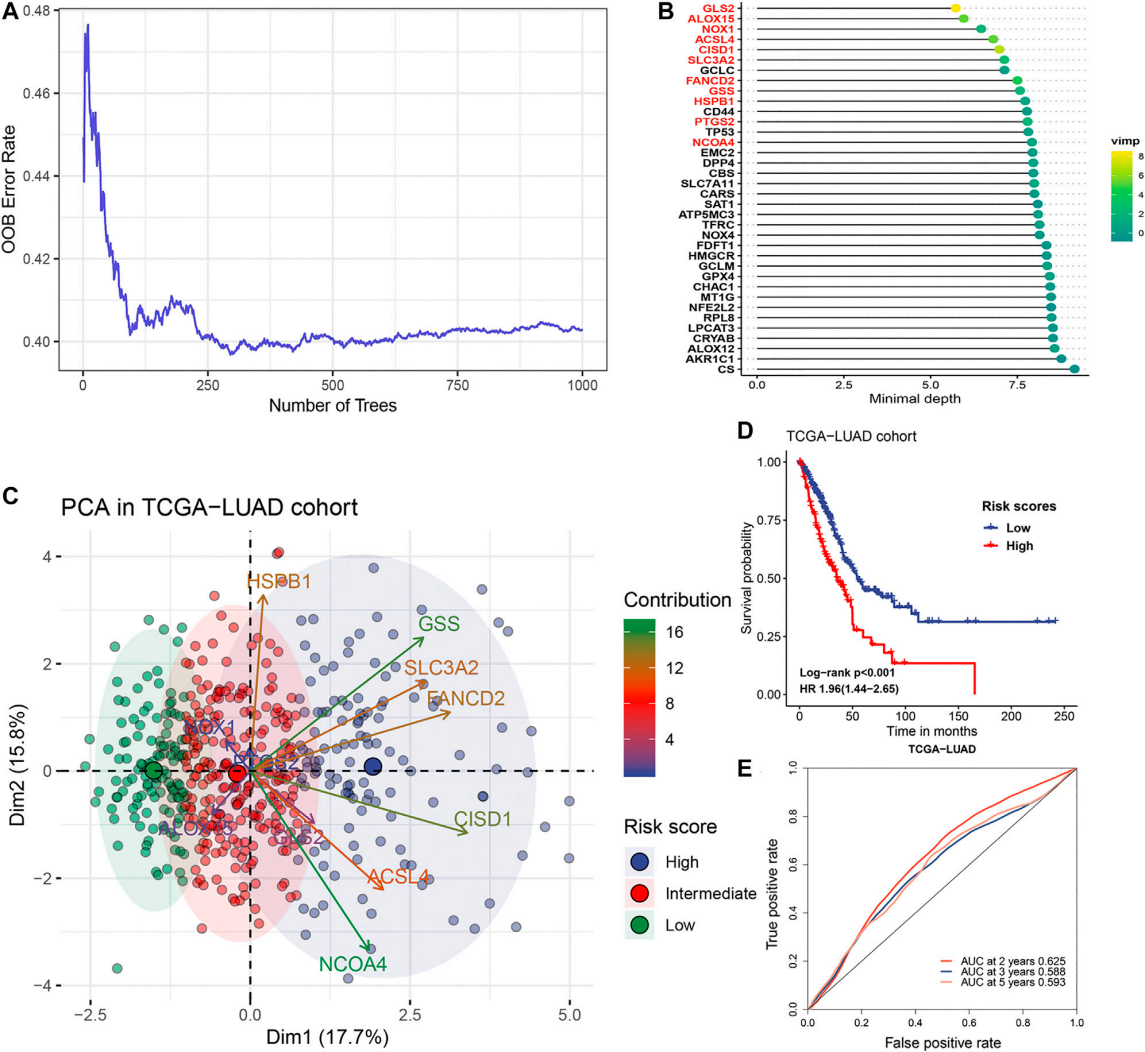
Gene selection and model construction process. **(A)** Estimation of the random forest OOB prediction error rate based on the number of trees. **(B)** The top 15 genes with the smallest minimal depth. VIMP is proportional to the size of the circle. **(C)** The distribution of risk scores. Gene contributions to PCA1 were annotated by gradient colors. The upper quartile and lower quartile of PCA1 were used for stratification. **(D)** Kaplan-Meier curves and univariate Cox regression of overall survival in the TCGA-LUAD cohort stratified by risk score. **(E)** ROC curve analysis for evaluating the prediction performance in the TCGA-LUAD cohort.

The flow chart for the analysis in this study is shown in Figure 1. Gene expression values of 36 ferroptosis regulators (Supplementary Table S1) served as input to construct the random survival forest model. Error plots using the out-of-bag (OOB) prediction error estimator illustrated that the forest prediction error tended to be stable when the number of trees was approximately 280 (Figure 2A). During the feature selection process, the top 15 genes ranked by minimal depth were listed, and 11 genes were chosen after considering VIMP (Figure 2B). Finally, GLS2, ALOX15, NOX1, ACSL4, CISD1, SLC3A2, FANCD2, GSS, HSPB1, PTGS2, and NCOA4 were used for further model construction. PCA was then conducted, and PC1 was the most significant component for prognostic prediction. Thus, PC1 served as FRRS, and the distribution of cases in TCGA-LUAD is illustrated in Figure 2C. Patients were classified into two subgroups based on an optimal cutoff risk score, which was calculated by the maximally selected rank statistics. Both the Kaplan-Meier survival curve and univariate Cox regression model demonstrated significantly (*p <* 0.001) worse overall survival in patients with higher FRRS (Figure 2D). The AUCs of the ROC curve were 0.625, 0.588, and 0.593 for 2, 3 and 5 years, respectively, (Figure 2E). Furthermore, multivariate Cox regression analysis indicated that T stage, lymph node metastasis and FRRS were independent prognostic factors (*p <* 0.001) (Table 2).

**TABLE 2:** Univariate and multivariate analyses of risk factors for prognosis in the TCGA-LUAD cohort.

	Univariate analysis			Multivariate analysis		
	P value	HR	95%CI	P value	HR	95%CI
Age						
≤66 years >66years	0.091	1.302	0.959–1.769			
Gender
Female Male	0.353	1.156	0.851–1.569			
Smoking						
Never Ever	0.985	0.996	0.640–1.549			
T stage						
I-II III-IV	**0.000**	2.313	1.557–3.436	**0.003**	1.920	1.246–2.959
lymph node metastasis						
Negative Positive	**0.000**	2.507	1.843–3.411	**0.000**	2.088	1.429–3.050
Distant metastasis						
no yes	**0.018**	1.188	1.030–1.369	0.283	1.091	0.931–1.278
TNM stage						
I-II III-IV	**0.000**	2.343	1.687–3.254	0.825	1.054	0.660–1.683
Risk score (median)						
low risk high risk	**0.000**	2.178	1.582–2.999	**0.000**	1.875	1.351–2.602

Bold indicates P < 0.05.

### Validating the Predictive Value of FRRS in Independent Cohorts

**FIGURE 3:**
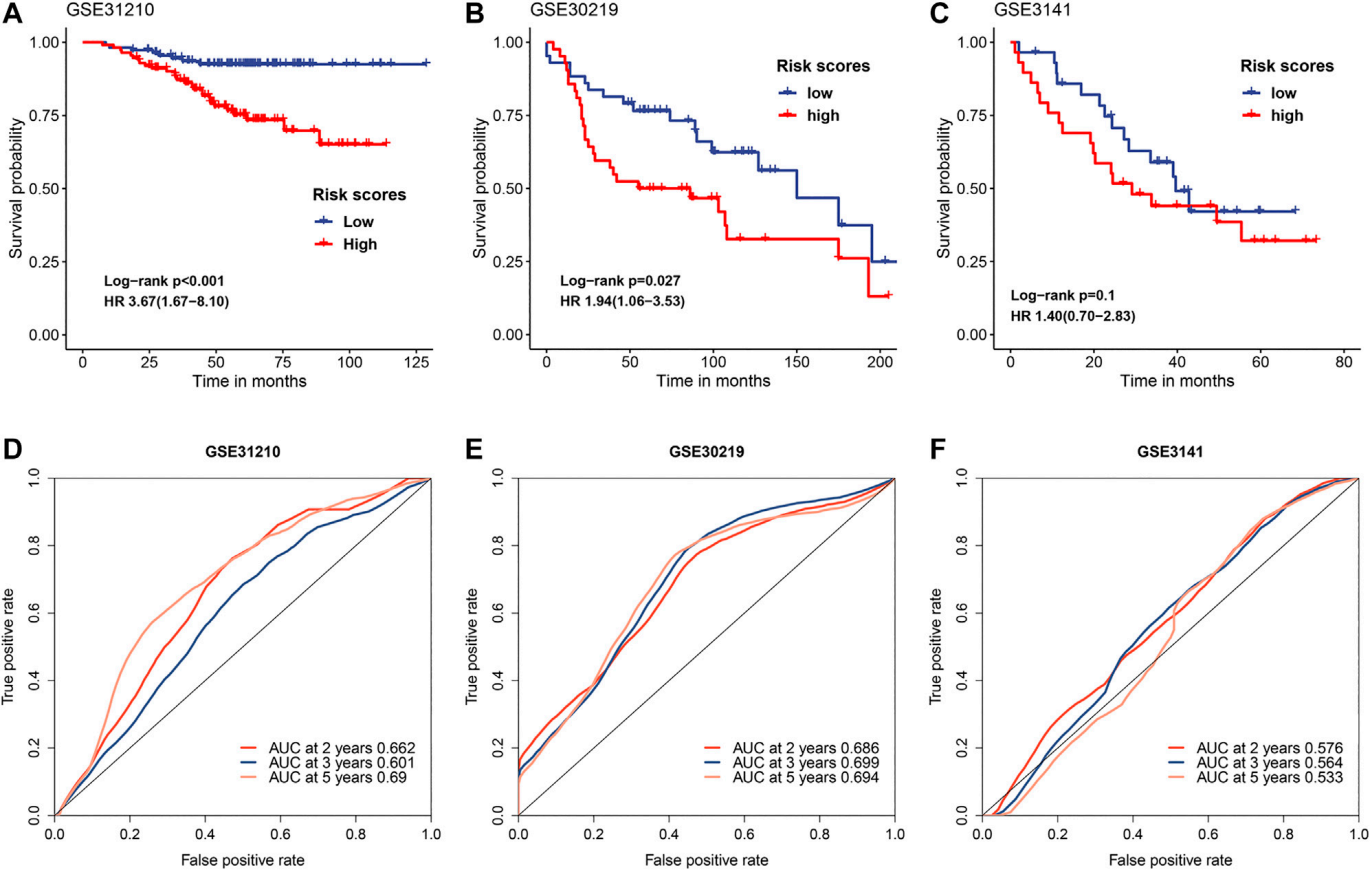
Evaluation of FRRS performance in independent datasets. Kaplan-Meier curves and univariate Cox regression of overall survival in **(A)** GSE31210, **(B)** GSE30219, and **(C)** GSE3141. ROC curve analyses in **(D)** GSE31210, **(E)** GSE30219, and **(F)** GSE3141.

To further verify the predictive value of FRRS, three independent cohorts from GEO were employed. Cox regression analysis and the Kaplan-Meier curve showed that worse overall survival was significantly (GSE31210 *p* < 0.001, GSE30219 *p* = 0.027) associated with higher FRRS in two independent cohorts (Figures 3A,B), which agreed with the results found in the TCGA-LUAD cohort. An exception was noted with GSE3141 (*p* = 0.100). Although not significant, a similar prognostic trend was observed (Figure 3C). The area under the ROC curves in GSE31210 was 0.662, 0.601 and 0.690 for 2, 3, and 5 years, respectively, (Figure 3D). In the GSE30219 cohort, the AUCs of FRRS at 2, 3 and 5 years were 0.686, 0.699, and 0.694, respectively, (Figure 3E). For the GSE3141 cohort, all AUCs were no more than 0.600 (Figure 3F).

### Expression Profile of 11 Selected Ferroptosis Regulators in the TCGA-LUAD Cohort

**FIGURE 4:**
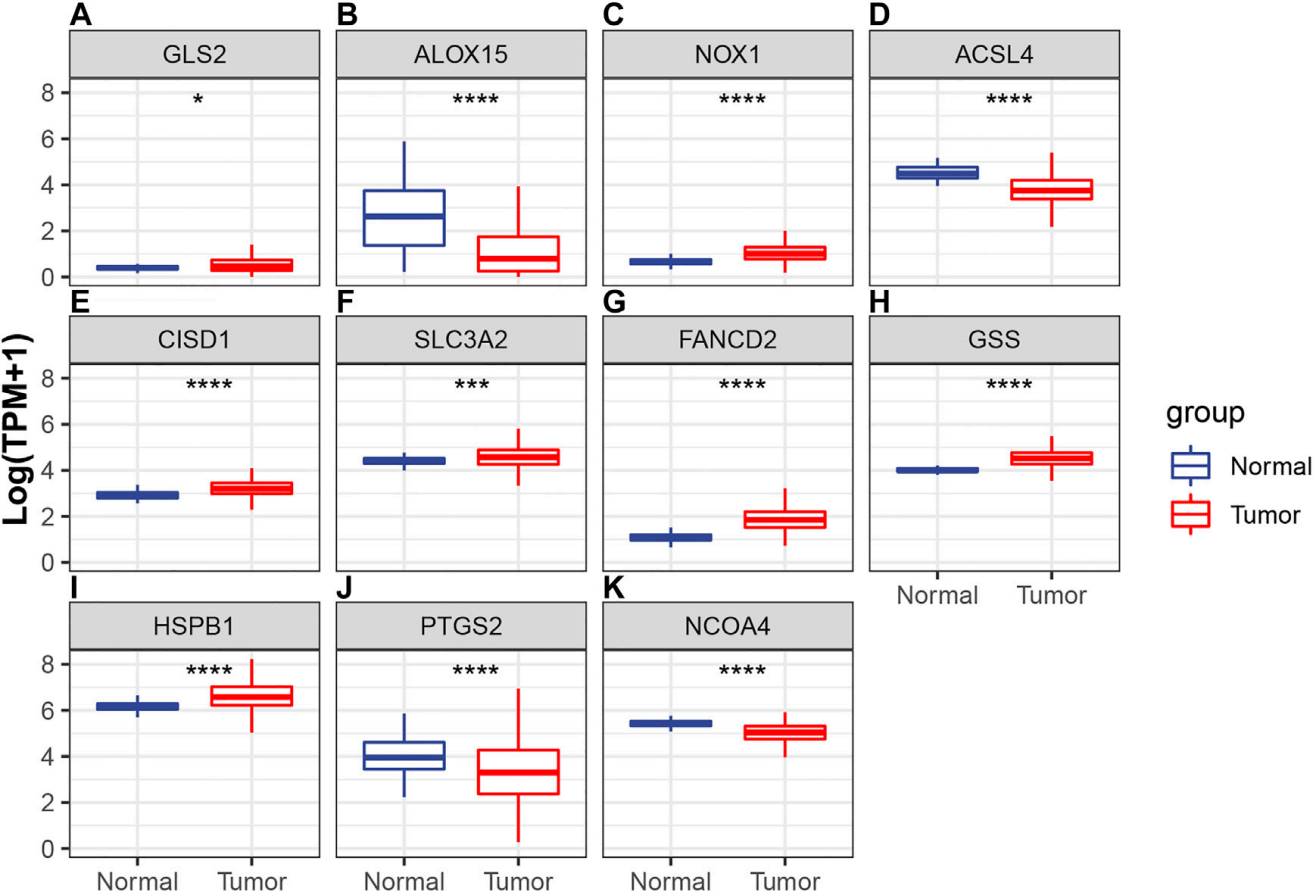
Comparisons of the expression profiles of 11 selected ferroptotic regulators between LUAD and normal tissue, which included **(A)** GLS2, **(B)** ALOX15, **(C)** NOX1, **(D)** ACSL4, **(E)** CISD1, **(F)** SLC3A2, **(G)** FANCD2, **(H)** GSS, **(I)** HSPB1, **(J)** PTGS2, and **(K)** NCOA4. *p < 0.05; ***p < 0.001; ****p < 0.0001.

**FIGURE 5:**
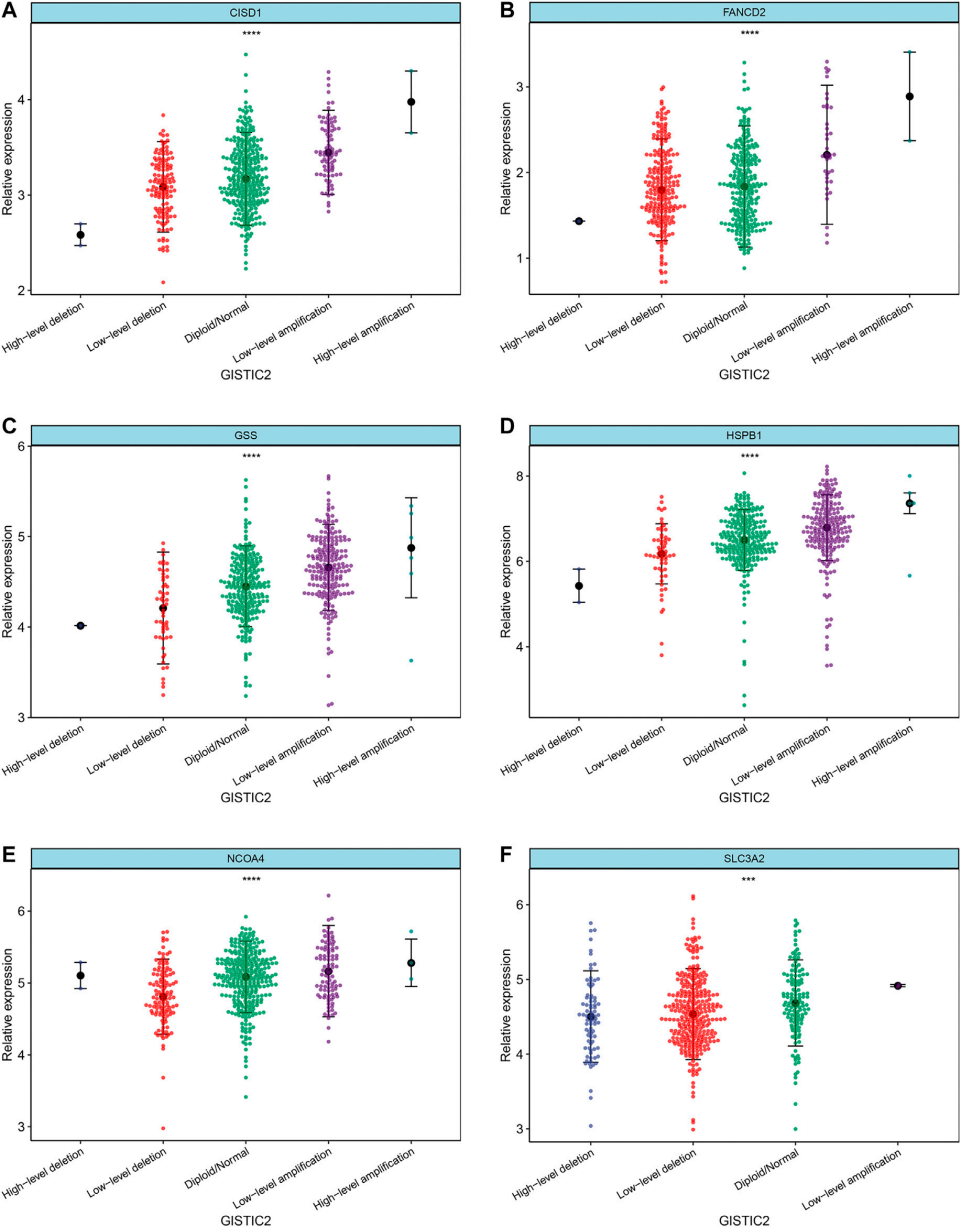
Correlation between copy number variation and mRNA expression of ferroptotic regulators, including **(A)** CISD1, **(B)** FANCD2, **(C)** GSS, **(D)** HSPB1, **(E)** NCOA4, and **(F)** SLC3A2. ***p < 0.001; ****p < 0.0001.

Differences in the expression levels of 11 selected ferroptosis regulators were compared between 526 tumor tissue samples and 59 adjacent normal tissue samples. Compared with expression in normal tissue, GLS2, NOX1, CISD1, SLC3A2, FANCD2, GSS, and HSPB1 expression levels were significantly increased, while expression levels of ALOX15, ACSL4, PTGS2, and NCOA4 were significantly decreased in LUAD (Figures 4A–K). Next, the implications of copy number variation on gene expression were analyzed. Only CISD1, FANCD2, GSS, HSPB1, NCOA4, and SLC3A2 revealed a significant association between copy number variation and gene expression (Figures 5A–F, Supplementary Figure S1). These results revealed that gene expression values may not be mainly regulated by copy number variations. Instead, they may be mainly influenced by mutation or epigenetic regulation, which needs to be further explored in the future.

### Differences in Genomic Mutation Profiles and TMB Between FRRS Subgroups

**FIGURE 6:**
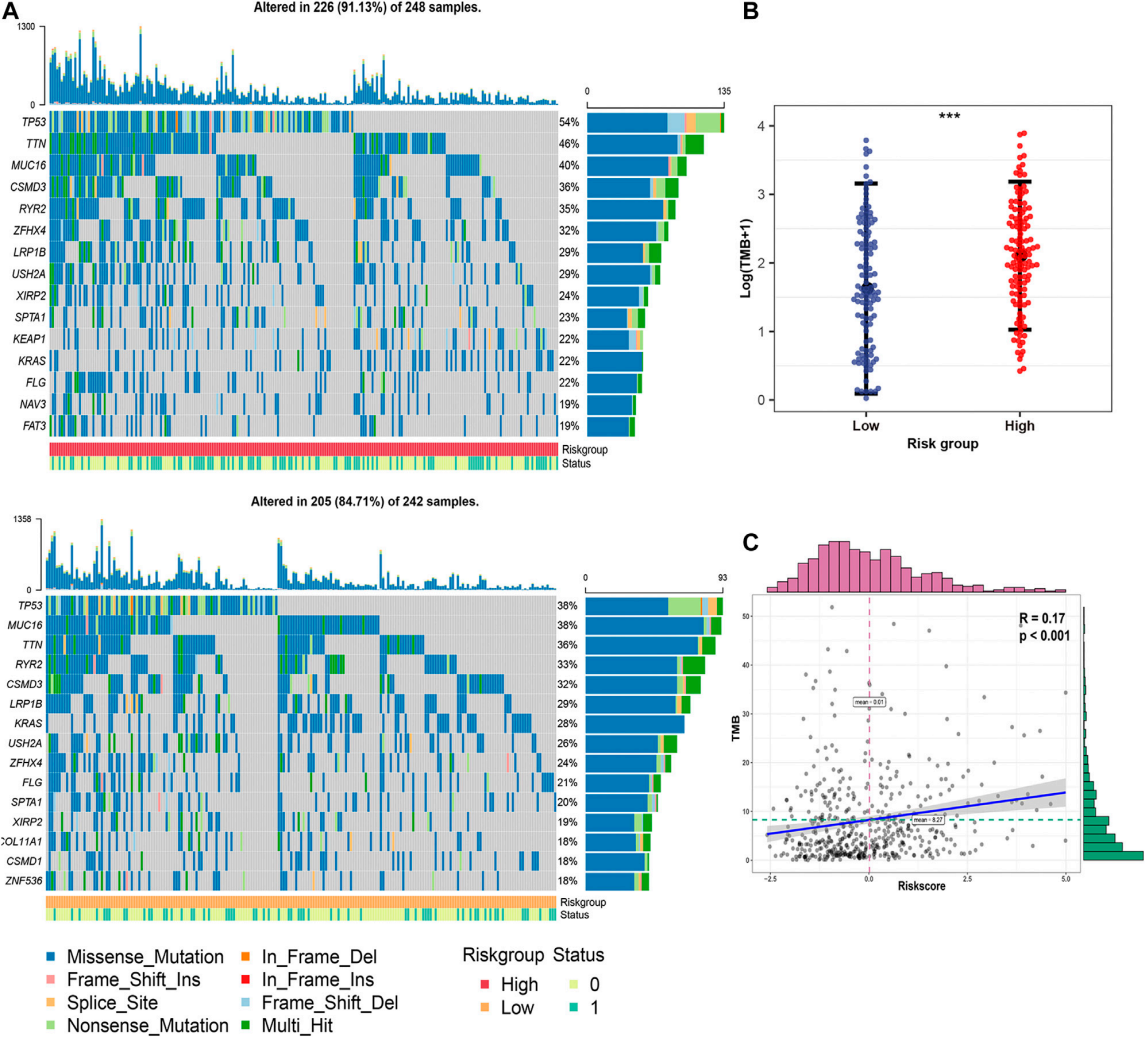
Genomic mutation profiles and TMB characteristics of different risk groups. **(A)** Distribution of frequently mutated genes in different TCGA-LUAD subgroups. The upper bar plot shows the TMB for each patient, whereas the left bar plot indicates the gene mutation frequency in different risk groups. The survival status of each patient is shown in the annotation bar. **(B)** Comparison of TMB between different subgroups in the TCGA-LUAD cohort. The whiskers embrace 1.5 times the interquartile range. ***p < 0.001. **(C)** Correlation analysis between TMB and FRRS in the TCGA-LUAD cohort.

The top 15 genes with the highest mutation frequency in different risk groups are shown in Figure 6A. Overall, the 12 top-ranked genes were shared between both sets (TP53, TTN, MUC16, CSMD3, RYR2, ZFHX4, LRP1B, USH2A, XIRP2, KRAS, SPTA1, and FLG). Three genes, including KEAP1, NAV3, and FAT3, were expressed only in the high-risk group, while COL11A1, CSMD1, and ZNF536 were specifically expressed in the low-risk group. Further correlation analysis suggested that TMB in the high-risk group was significantly (*p* < 0.001) higher than that in the low-risk group (Figure 6B). In addition, a significant (*p* < 0.001) positive correlation was observed between FRRS and TMB (Figure 6C).

### Functional Annotation and Pathway Enrichment Analysis of FRRS

**FIGURE 7:**
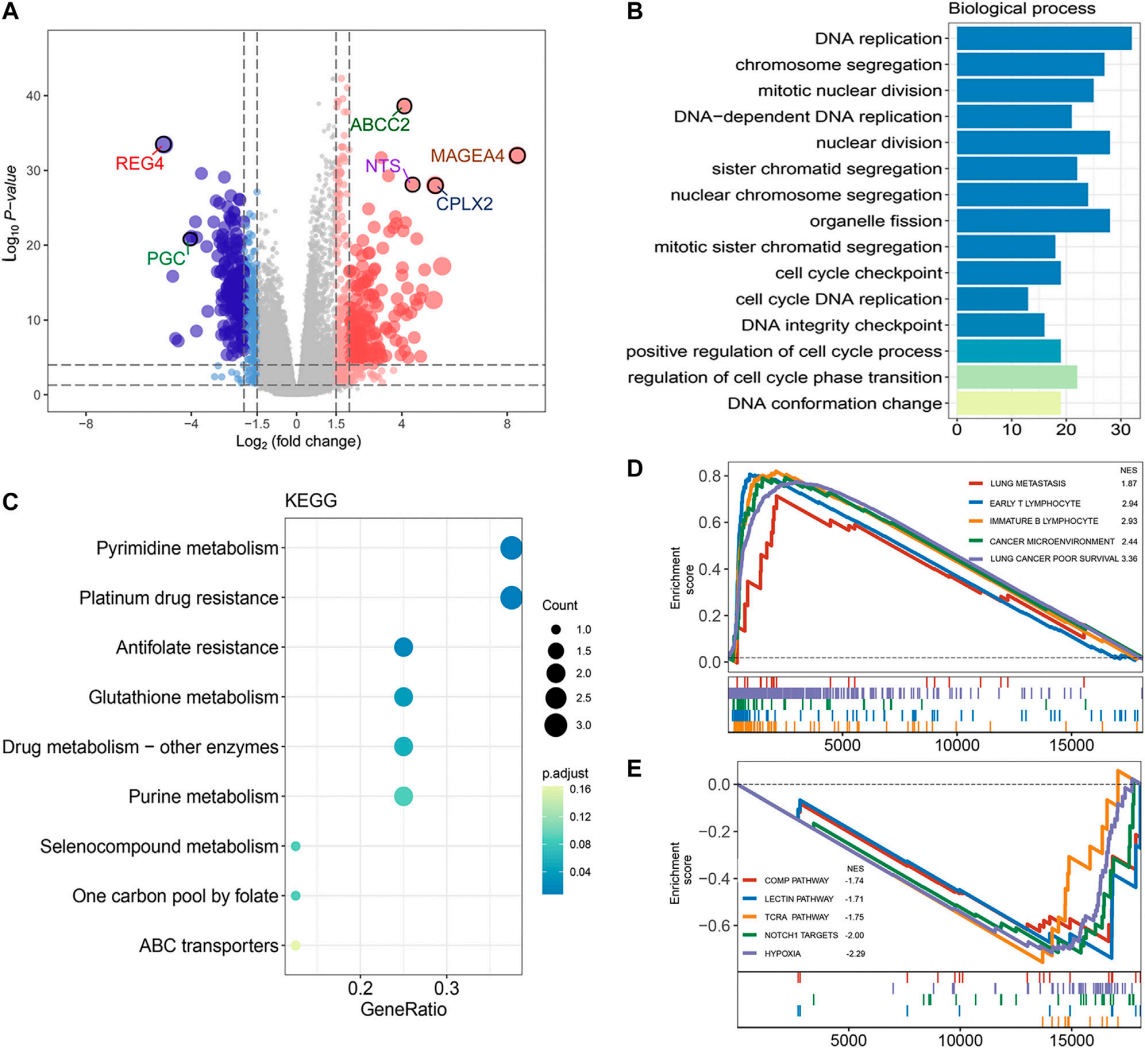
Functional annotation and pathway enrichment analysis of FRRS. **(A)** Volcano plot of differential gene expression analysis between high-risk and low-risk groups. The upper quartile and lower quartile of FRRS were used for stratification. The size of points is positively correlated with lower *p* value and higher fold change. **(B)** Functional annotation for FRRS using GO biological process. The color scale of bar plots indicates the significance level of enrichment. **(C)** Pathway enrichment analysis by KEGG. The color scale of circles represents the significance level of enrichment. **(D, E)** Gene set enrichment analysis of curated gene sets obtained from MSigDB Collections. Pathways of interest with significant enrichment between high-risk and low-risk groups are shown.

To identify the potential biological function of FRRS, both the DEG-based method and GSEA were conducted. Using the edgeR package, 664 genes were shown (Figure 7A) to be differentially expressed based on our predefined criteria (adjusted *p* < 0.01 and |log_2_ (fold change)| > 2). Significantly annotated biological processes and KEGG pathways of these DEGs are summarized in Figures 7B,C, respectively. Functional enrichment analysis was then performed by implementing the GSEA algorithm, which took all genes into account. Annotated pathways of interest are displayed in Figures 7D,E. The enrichment results illustrated that processes related to poor survival in lung cancer patients, cancer microenvironment, immature B lymphocytes, early T lymphocytes and lung metastasis were significantly enriched in the high-risk group (Figure 7D), while processes related to COMP, lectin, TCRA, NOTCH1 target and hypoxia were significantly enriched in the low-risk group (Figure 7E). A full list of GSEA results can be found in Supplementary Table S2.

### Differential Levels of Specific Emerging Immunotherapy Targets and Immune Cell Abundances Between FRRS Subgroups

**FIGURE 8:**
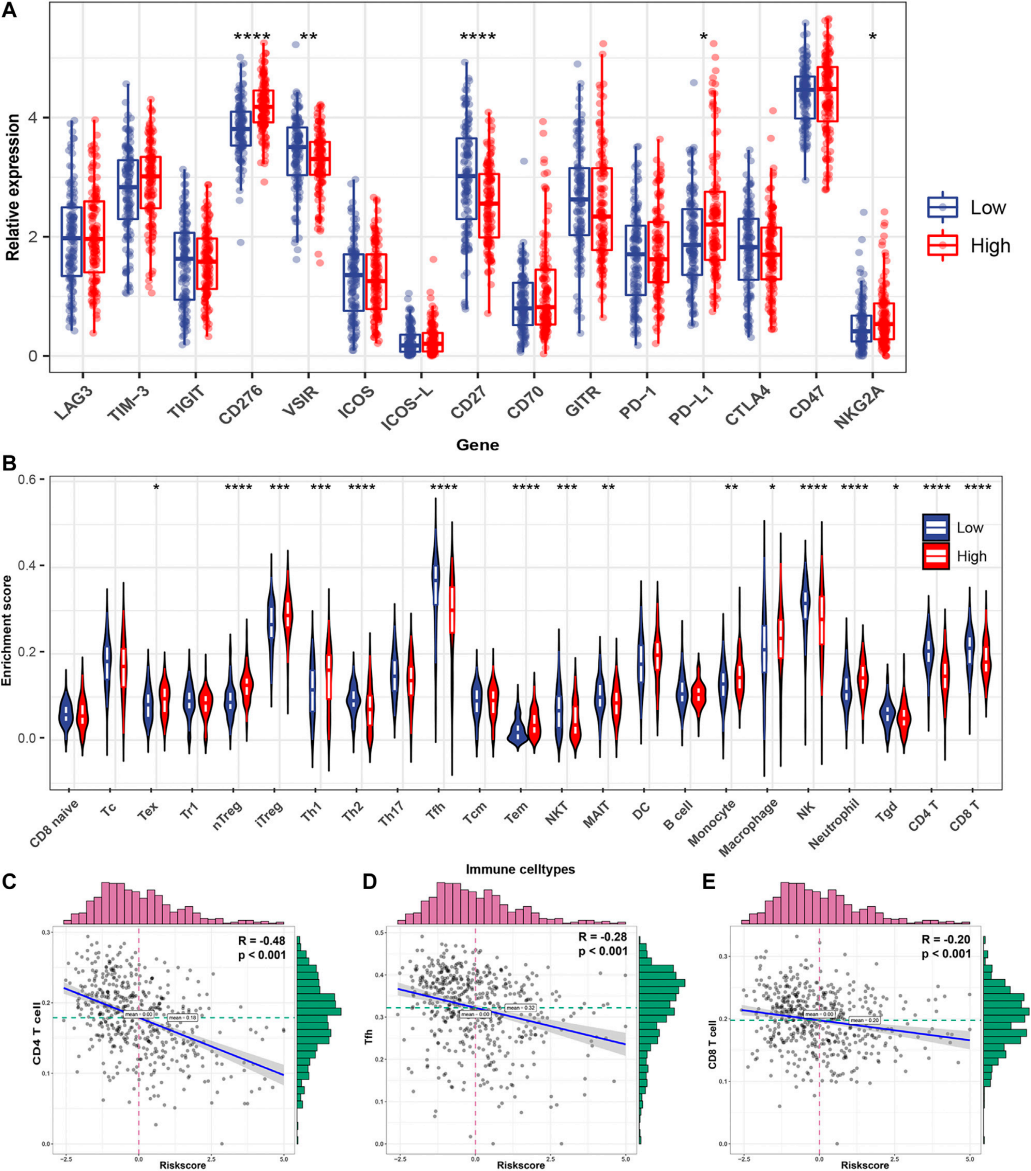
Immune characteristics of the TCGA-LUAD subtype. **(A)** Different expression of available emerging immunotherapeutic targets between high-risk and low-risk groups. **(B)** Different infiltrating abundances of 24 immune cell types estimated by ImmuCellAI between subgroups. Correlation analysis between FRRS and **(C)** CD4 T cells, **(D)** Tfhs, and **(E)** CD8 T cells. The distributions of FRRS and infiltrating of immune cell abundance are shown on the top and right of the plot, respectively. *p < 0.05; **p < 0.01; ***p < 0.001; ****p < 0.0001.

Pathway enrichment in the previous section suggested that FRRS is involved in several immune-signaling pathways. Thus, specific association analyses between FRRS and several immune components were carried out. The gene expression levels of potential immunotherapy targets, including CD276 (*p* < 0.001), PD-L1 (*p* = 0.011), and NKG2A (*p* = 0.045), were significantly upregulated in the high-risk group (Figure 8A). Meanwhile, the expression levels of VSIR (*p* = 0.009) and CD27 (*p* < 0.001) were significantly higher in the low-risk group than in the high-risk group (Figure 8A). In terms of infiltrating immune cells, among the high-risk group, the abundances of Tex (*p* = 0.012), nTreg (*p* < 0.001), iTreg (*p* < 0.001), Th1 (*p* < 0.001), Tem (*p* < 0.001), monocytes (*p* = 0.001), macrophages (*p* = 0.023) and neutrophils (*p* < 0.001) were significantly higher than those in the low-risk group (Figure 8B). The results also revealed that the abundances of CD4 T (*p* < 0.001), CD8 T (*p* < 0.001), Th2 (*p* < 0.001), Tfh (*p* < 0.001), NKT (*p* < 0.001), MAIT (*p* = 0.009), NK (*p* < 0.001), and Tgd (*p* = 0.014) cells were significantly lower in the high-risk group than in the low-risk group (Figure 8B). The top three immune cell types with the highest correlation with FRRS were CD4 T (R = ˗0.48, *p* < 0.001), Tfh (R = ˗0.28, *p* < 0.001), and CD8 T cells (R = -0.20, *p* < 0.001) (Figures 8C–E).

### Three Ferroptosis Regulators in FRRS are Potential Predictors of Immunotherapeutic Response

**FIGURE 9:**
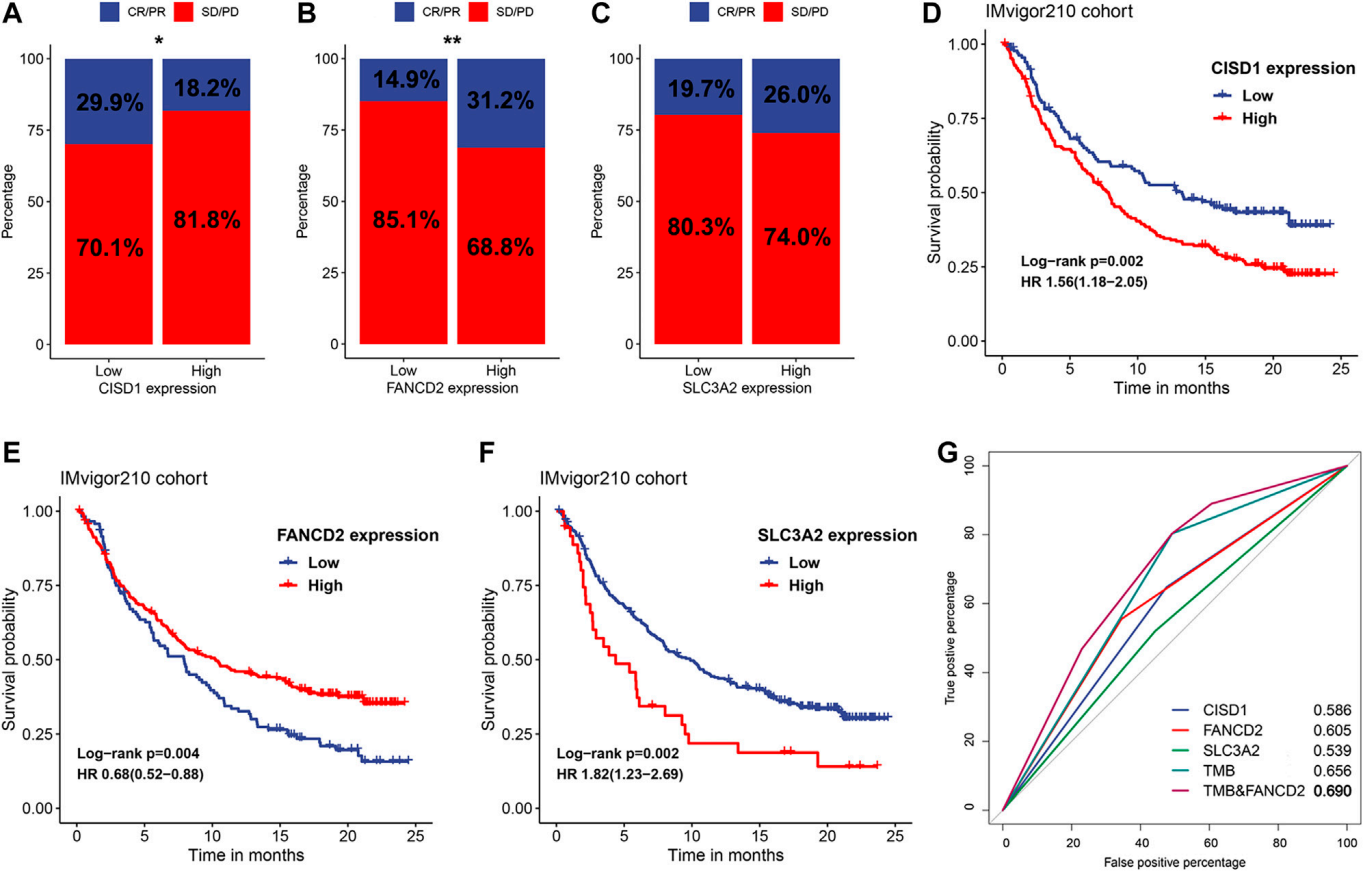
Identification of potential ferroptosis-based biomarkers for predicting therapeutic response. The proportion of response to anti-PD-L1 immunotherapy in subgroups stratified by the expression of **(A)** CISD1, **(B)** FANCD2, and **(C)** SLC3A2 in the IMvigor210 cohort. *p < 0.05; **p < 0.01. Kaplan-Meier curves and univariate Cox regression of patients with high and low expression of **(D)** CISD1, **(E)** FANCD2, and **(F)** SLC3A2 in the IMvigor210 cohort. **(G)** ROC curves evaluating the predictive values of CISD1, FANCD2, SLC3A2, TMB and the combination of FANCD2 and TMB in the IMvigor210 cohort.

Since close relationships were observed between FRRS and various immune components, exploration of the predictive values of ferroptosis regulators was then performed. The top three genes that contributed most to FRRS were CISD1, FANCD2 and SLC3A2, and their expression values were extracted from IMvigor210 RNA-seq data. The results illustrated that low CISD1 expression was significantly (*p* = 0.027) associated with favorable immunotherapeutic responses, including complete response (CR) and partial response (PR) (Figure 9A). In contrast, low expression of FANCD2 was significantly (*p* = 0.001) associated with undesired effects, including stable disease (SD) and progressive disease (PD) (Figure 9B). No significant association was observed between SLC3A2 expression and therapeutic effect (Figure 9C). The Kaplan-Meier survival curve showed that high expression of CISD1 (*p* = 0.002) and SLC3A2 (*p* = 0.002) was associated with a significantly poorer overall survival, and high expression of FANCD2 conferred a significant (*p* = 0.004) favorable prognosis (Figures 9D,E). The AUCs of FRRS at 2, 3 and, 5 years were 0.686, 0.699, and 0.694, respectively. Using CISD1, FANCD2, SLC3A2, and TMB as predictors of immunotherapeutic response, their AUCs were 0.586, 0.605, 0.539, and 0.656, respectively, (Figure 9F). In addition, combining FANCD2 and TMB substantially increased predictive precision, and the value of AUC reached 0.690 (Figure 9F).

### Validating Ferroptosis Regulator Expression and the Predictive Value of FRRS in CPTAC-LUAD Cohorts

**FIGURE 10:**
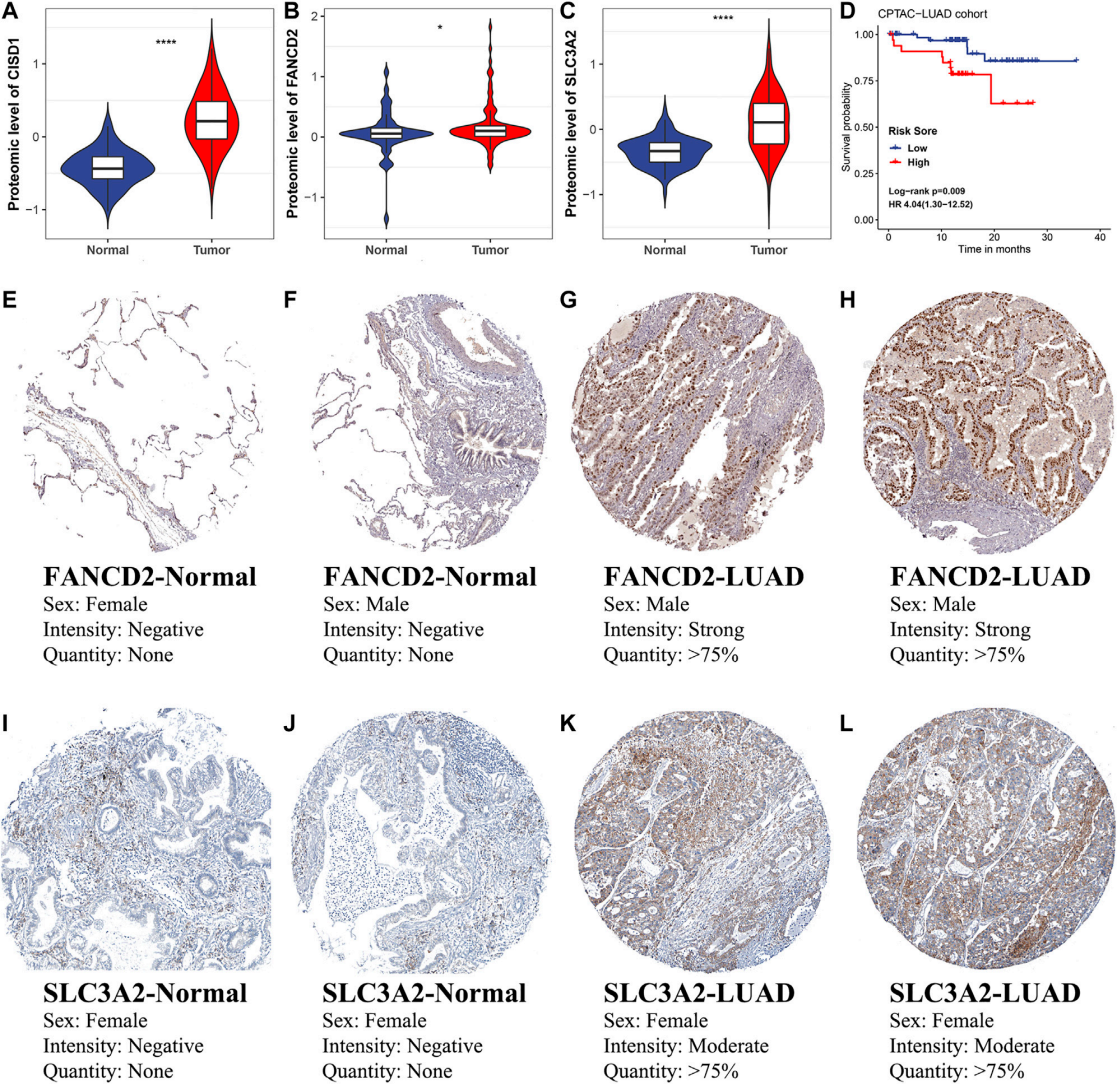
Protein-level validation of selected ferroptosis regulators and FRRS. Comparisons of **(A)** CISD1, **(B)** FANCD2 and **(C)** SLC3A2 protein expression between LUAD and normal tissues in the CPTAC-LUAD cohort. The unit of the y-axis is the unshared log ratio. *p < 0.05; ****p < 0.0001. **(D)** Kaplan-Meier curves and univariate Cox regression of overall survival in the CPTAC-LUAD cohort. **(E–H)** Immunohistochemical staining for FANCD2 in normal lung tissue **(E, F)** and LUAD **(G, H)**. **(I–L)** Immunohistochemical staining for SLC3A2 in normal lung tissue **(I, J)** and LUAD **(K, L)**.

To further assess the clinical significance of ferroptosis regulators and FRRS, protein-level validation was conducted. The expression of CISD1 (*p* < 0.001), FANCD2 (*p* = 0.036) and SLC3A2 (*p* < 0.001) in tumors was significantly higher than that in adjacent normal tissues (Figures 10A–C). In addition, high FRRS was significantly (*p* = 0.009) associated with poor prognosis (Figure 10D). Immunohistochemical images of FANCD2 and SLC3A2 in both normal lung tissue and LUAD are shown in Figure 10E-L, which indicated expression of these proteins in patient tissues. The immunohistochemical image of CISD1 was not displayed due to a lack of staining in LUAD.

## Discussion

Apoptosis has been identified as a core process regulating cell proliferation and as an important mechanism to prevent tumorigenesis (Wong, 2011). Ferroptosis, one form of programmed cell death that is independent of apoptosis, has been modulated by several pathways and has been implicated in various diseases (Stockwell et al., 2020). Specifically, several studies have demonstrated that ferroptosis is closely involved in cancer development, the tumor suppression process and the treatment response (Lu et al., 2018). However, research on ferroptosis in oncology is still in its infancy, especially in LUAD. In this study, the integrative roles of 36 ferroptosis regulators were analyzed, and a ferroptosis-based score was constructed to predict prognosis in four datasets. In addition, three ferroptosis regulators showed value in predicting therapeutic response and are worthy of further investigation. It is important to note that the datasets included in our study were heterogeneous with respect to tumor stage, which led to a significant difference in overall median survival.

With regard to model construction, instead of using the most common least absolute shrinkage and selection operator (LASSO), a random survival forest algorithm was applied for feature reduction for the following reasons. First, two forms of randomization were incorporated, including case resampling and variable subsetting, which made the prediction results robust and accurate (Ishwaran et al., 2008). Second, the inherent attributes of ensemble learning make it easy to handle both nonlinear effects and variable interactions. Third, two tested quantitative indicators, VIMP and minimal depth, could be used for gene selection. Nevertheless, the disadvantages of the random survival forest were also apparent. It did not provide explicitly formatted formulas and could not extract information regarding the underlying process. Using such a model in a clinical setting without knowing its biological explanation may be dangerous. Thus, the random survival forest was only implemented for gene selection, and PCA was applied for scoring. Currently, several algorithms can estimate the abundance of infiltrating immune cells from bulk transcriptome data. However, only ImmuCellAI is designed to predict the abundance of numerous T-cell subsets, which are key elements in immunotherapy. Moreover, the abundance of most immune cells estimated by ImmuCellAI showed a higher positive correlation with the counting results of flow cytometry than that estimated by the other methods, particularly for T-cell subsets. According to our current understanding, ferroptosis regulatory genes can be grouped into three main metabolic pathways: amino acid metabolism, lipid metabolism and iron metabolism (Gao et al., 2015; Yang et al., 2016; Angeli et al., 2017). Ferroptosis is at the intersection of these pathways, and its activity depends on the balance of both inhibitory and activating metabolites. In our present study, functional enrichment analysis of FRRS results identified several metabolic pathways that coincided well with previous *in vitro* and *in vivo* results. In the context of cancer, several conceivable mechanisms that modulate ferroptosis sensitivity have begun to emerge recently. These mechanisms are mainly involved in tumor suppression genes, hypoxia inducible factors and the degree to which cells were in a mesenchymal state (Jiang et al., 2015; Viswanathan et al., 2017; Zou et al., 2019). The mesenchymal-like state is attractive in research since cancer cells at this state are treatment-resistant and possess stem cell-like features (Viswanathan et al., 2017). Meanwhile, Richard et al. pointed out that cells with mesenchymal-like features are also resistant to some types of targeted therapy (Richard et al., 2016). Together, these factors may be a major cause of treatment failure for most cancer patients. However, numerous studies have reported that mesenchymal-like cancer cells are associated with an inherent sensitivity to ferroptosis, and we also reported significant enrichment of ferroptosis in platinum drug resistance and drug metabolism. This has brought novel strategies and targets to overcome drug resistance.

Based on a literature review, we included 36 ferroptosis regulators in this study and extracted 11 genes for comprehensive analysis. It is worth noting that all selected ferroptosis regulators were significantly different in their expression between LUAD tissue and normal tissue, which indicated a fundamental change in ferroptosis activity. Specifically, the expression of two important ferroptosis activators, ACSL4 and NCOA4, was significantly decreased (Mancias et al., 2014; Doll et al., 2017). ACSL4 is a synthase that converts free fatty acids into peroxidation products, which is required for ferroptosis (Doll et al., 2017). On the other hand, NCOA4 participates in regulating ferritinophagy and controlling the abundance of iron (Mancias et al., 2014). While indirect, these results provide evidence of ferroptosis dysregulation in LUAD. Whether reactivation of ferroptosis can impede LUAD occurrence and development remains to be further investigated.

Numerous previous studies have demonstrated that ferroptosis is involved in immunomodulation and immune evasion. There is extensive crosstalk between ferroptosis in cancer cells and infiltrating immune cells. Distinct signals released by ferroptotic cancer cells can trigger phagocytosis and stimulate antigen presentation by dendritic cells (Angeli et al., 2019). Meanwhile, *in vivo* suppression of ferroptosis activity results in impediment of cell killing ability for both CD8 T cell and NK cells (Böttcher et al., 2018; Wang et al., 2019). Similar to the aforementioned studies, ferroptosis was found to be associated with several immune-related pathways. Meanwhile, FRRS was significantly correlated with CD8 T cell abundance and TMB. Because of these results, we investigated the potential for predicting therapeutic responses in relation to these regulators. In the IMvigor210 cohort, a combination of FANCD2 and TMB improved the predictive efficiency to a certain extent. Several previous studies have shown that FANCD2 is closely associated with several cancer types, including breast cancer, hepatocellular carcinoma and leukemia (Lewis et al., 2005; Yao et al., 2015; Komatsu et al., 2017). In lung cancer, Wang et al. also reported that inhibition of FANCD2 enhanced DNA damage and restrained tumor progression (Wang et al., 2015). Unfortunately, this IMvigor210 cohort only included patients with urothelial cancer. However, with a deeper understanding of tumor genomics, tumors from different sites may share similar genomic alternation profiles and can be treated with the same drugs or be predicted with the same indicators. Therefore, findings in urothelial carcinoma may also have implications in other solid tumors, including LUAD, to some extent.

This preliminary study has several limitations. First, since the study of ferroptosis is a novel and rapidly evolving field, an increasing number of ferroptosis regulators will likely be found, not just the 36 genes included in the present study. Second, the predictive effectiveness was relatively modest due to the limited number of features. The model can be further optimized after a deeper understanding of the biological processes of ferroptosis. Third, all quantifications of gene expression are relative values, which makes it difficult to determine the absolute threshold in clinical application. Thus, a large-scale LUAD cohort with absolute quantitative measurements of gene expression is required to further verify the conclusion.

In summary, this is the first report of ferroptosis in an LUAD cohort. The present study has profiled significant alternations of ferroptosis regulators in LUAD, which may open up novel possible drug targets against LUAD. A robust ferroptosis-based prognostic risk score named FRRS was constructed and validated in independent cohorts. More importantly, FRRS was found to be significantly associated with several immune components, including CD8 T cells, that are closely related to the antitumor response. Combining data concerning FANCD2 and TMB could further improve performance in predicting the immunotherapeutic response. Finally, protein-level validation of FRRS was conducted through proteomics and immunohistochemistry to demonstrate its clinical utility. This preliminary study provides a novel stratification system for LUAD patients and reveals potential predictive biomarkers for immunotherapy response.

## Data Availability

The datasets presented in this study can be found in online repositories. The names of the repository/repositories and accession number(s) can be found in the article/Supplementary Material.
