# Supplementary figures and images for: Comprehensive Analysis of Ferroptosis Regulators in Lung Adenocarcinomas Identifies Prognostic and Immunotherapy-Related Biomarkers
Source: Front Mol Biosci. 2021 Mar 12;8:587436. doi: 10.3389/fmolb.2021.587436 (PMC7994623; doi:10.3389/fmolb.2021.587436)

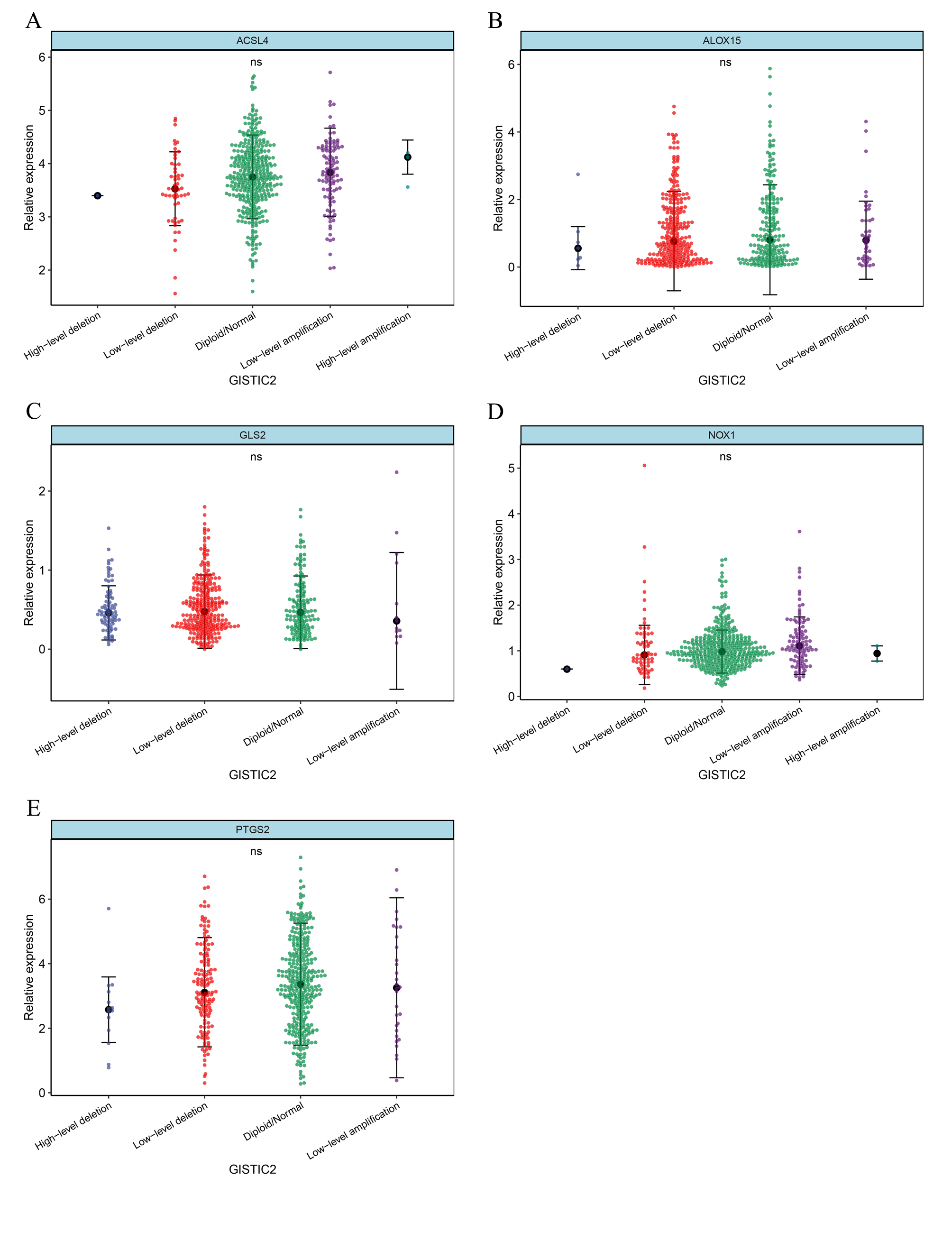

Supplement: Supplementary file 1 [file image1.tif]
