# Supplementary material for: Comprehensive Analysis of Ferroptosis Regulators in Lung Adenocarcinomas Identifies Prognostic and Immunotherapy-Related Biomarkers
Source: Front Mol Biosci. 2021 Mar 12;8:587436. doi: 10.3389/fmolb.2021.587436 (PMC7994623; doi:10.3389/fmolb.2021.587436)
Supplement: Supplementary file 3 [file table2.docx]

Table 2 Univariate analysis and multivariate analysis of risk factors for prognosis in TCGA-LUAD cohort

|  | Univariate analysis | | | Multivariate analysis | | |
| --- | --- | --- | --- | --- | --- | --- |
|  | P value | HR | 95%CI | P value | HR | 95%CI |
| **Age**  ≤66 years  >66years | 0.091 | 1.302 | 0.959-1.769 |  |  |  |
| **Gender**  female  male | 0.353 | 1.156 | 0.851-1.569 |  |  |  |
| **Smoking**  never  ever | 0.985 | 0.996 | 0.640-1.549 |  |  |  |
| **T stage**  I-II  III-IV | **0.000** | 2.313 | 1.557-3.436 | **0.003** | 1.920 | 1.246-2.959 |
| **lymph node metastasis**  negative  positive | **0.000** | 2.507 | 1.843-3.411 | **0.000** | 2.088 | 1.429-3.050 |
| **Distant metastasis** |  |  |  |  |  |  |
| no  yes | **0.018** | 1.188 | 1.030-1.369 | 0.283 | 1.091 | 0.931-1.278 |
| **TNM stage**  I-II  III-IV | **0.000** | 2.343 | 1.687-3.254 | 0.825 | 1.054 | 0.660-1.683 |
| **Risk score (median)**  low risk  high risk | **0.000** | 2.178 | 1.582-2.999 | **0.000** | 1.875 | 1.351-2.602 |

* Bold indicates *P* < 0.05.
